# Stability of Network Models Linking Personality to Conspiracy Mentality Before and During the COVID-19 Pandemic

**DOI:** 10.5964/ejop.14761

**Published:** 2026-02-27

**Authors:** Lukasz Stasielowicz

**Affiliations:** 1 Institute of Psychology, Osnabrück University, Osnabrück, Germany; University of Western Australia, Perth, Australia

**Keywords:** conspiracy mentality, conspiracy beliefs, personality, network models, COVID-19

## Abstract

Discussions about potential intervention targets, antecedents, and consequences of conspiracy beliefs often rely on comparing bivariate correlations, which can mask intricate patterns. Therefore, the present study adopts a multivariate network approach to gain nuanced insights into the relationships between personality variables and conspiracy mentality. Established and less-studied correlates of conspiracy mentality (i.e., bedtime procrastination, life satisfaction, locus of control, neuroticism, political cynicism, self-efficacy, and self-esteem) were examined together in network models at the aggregate score level and item level. Notably, network stability was examined across different samples before (*N* = 403) and during (*N* = 193) the COVID-19 pandemic. The main findings are: (a) the strength and sign of the relationships were often stable across bivariate and network analyses (e.g., positive relationships between political cynicism and conspiracy mentality), however, there were exceptions, such as an inconsistent link between life satisfaction and conspiracy mentality; (b) while many network relationships and centrality indices were similar before and during the COVID-19 pandemic, some noteworthy exceptions indicate that interventions targeting implausible conspiracy beliefs may benefit from tailoring to external circumstances; (c) certain influential network elements were identified that could inform future interventions (e.g., increasing politicians' transparency and accountability).

According to available evidence (Swami, 2012; Swami et al., 2011), people with specific personality characteristics, such as political cynicism, are more likely to believe in conspiracy theories. Despite meta-analytic advances in identifying potentially relevant personality aspects (Biddlestone et al., 2025; Bowes et al., 2023; Hornsey et al., 2023; Stasielowicz, 2022b), various remaining research gaps limit our understanding of conspiracy beliefs. It is often unclear how the correlational evidence based on average personality and conspiracy belief scores can translate into specific intervention recommendations. Since traditional meta-analytic techniques cannot estimate the unique contributions of particular personality aspects to conspiracy beliefs, the present study aims to advance the research field by showcasing how an underutilized multivariate approach can be harnessed to offer a comprehensive perspective on conspiracy beliefs and potentially point out new research directions. Specifically, the present study investigates networks consisting of conspiracy mentality and personality variables.

Hitherto, the network approach was rarely adopted when examining conspiracy beliefs (Williams et al., 2022). However, network analyses can help identify the central aspects (Costantini et al., 2015) linking conspiracy mentality and personality. Elucidating which aspects of conspiracy mentality are connected to the highest number of personality variables can inform future interventions. Furthermore, the network approach helps establish which connections are the strongest. Those central elements of the network might be good intervention targets (Robinaugh et al., 2016). After all, targeting strongly connected aspects of conspiracy mentality could have positive spillover effects. Importantly, such focused interventions could help reduce intervention time, burden, and costs. Although cross-sectional network analyses cannot provide definitive causal evidence, the respective findings can, aligning with the goals of exploratory research (Ditroilo et al., 2025), generate empirically informed hypotheses. These hypotheses could then be used in future longitudinal and experimental studies to rigorously evaluate the potential of harnessing the identified patterns to advance the development of interventions.

Honing our understanding of the mechanisms underlying conspiracy mentality is required to increase the effectiveness of interventions aimed at reducing conspiracy beliefs. According to recent review articles (O’Mahony et al., 2023; Stasielowicz, 2026a), intervention effects tend to be small (e.g., *g* = 0.20). One option to move the research field forward is to quantify the effects of intervention techniques that have not yet been investigated on a large scale in the context of conspiracy beliefs, such as psychotherapy and chatbots (Costello et al., 2025; Wang & Tanes-Ehle, 2023). However, there is no guarantee that these new techniques will be more effective than traditional intervention techniques, such as fact-checking conspiracy claims. Therefore, it may be worth pursuing other research avenues. For example, one could strengthen the theoretical understanding of the mechanisms underlying conspiracy beliefs by providing empirical evidence about the relationships among various personality aspects and their relationships with conspiracy beliefs. This knowledge could then be used to tailor the language and framing of intervention materials to the participant's personality.

Although available meta-analytic findings provide a helpful overview of potentially relevant personality aspects (Biddlestone et al., 2025; Bowes et al., 2023; Hornsey et al., 2023; Stasielowicz, 2022b), it is impossible to account for all these variables in a single intervention study. Moreover, choosing specific personality variables is difficult, as it is unclear whether different personality variables uniquely contribute to conspiracy beliefs or if they are largely redundant. To help close this research gap and offer a nuanced perspective, the present study will simultaneously highlight the intricate relationships between various novel and frequently examined correlates of conspiracy beliefs.

Notably, the current study takes advantage of the unique opportunity to investigate the stability of the findings across different circumstances, as data collection was made before and during the COVID-19 pandemic. Examining network stability is important, as this step may help researchers decide whether developing particular one-size-fits-all interventions is promising or doomed to fail. Without ensuring the stability of the relationships, deployed interventions aimed at reducing conspiracy beliefs may be far less effective than assumed. If relationships with conspiracy beliefs are inconsistent, then tailoring interventions to sample characteristics or circumstances may be necessary.

## Present Study

Conspiracy theories can be defined (Nera & Schöpfer, 2023) as claims that “the public is being pervasively lied to regarding some aspect(s) of reality, to allow some group(s) to enact a harmful, self-serving agenda”. The current study focuses on the general tendency to believe in conspiracy theories — conspiracy mentality.

The focus on a conspiracy mentality rather than specific conspiracy theories, such as COVID-19 conspiracy theories, climate change conspiracy theories, and ethnicity-related conspiracy theories, is motivated by several factors. First, while measuring beliefs about multiple specific conspiracy theories requires using many questions, the Conspiracy Mentality Questionnaire is a five-item measure (Bruder et al., 2013), which ensures that participant burden is relatively low. Second, including fewer items reduces model complexity, which can facilitate the estimation of the relationships between conspiracy mentality and personality. Third, the relationships with personality variables tend to be very similar, irrespective of which questionnaire is used to assess conspiracy beliefs (Goreis & Voracek, 2019; Stasielowicz, 2022b). Thus, results based on a conspiracy mentality measure can inform research devoted to specific conspiracy theories. Fourth, although the conspiracy mentality is regarded as a one-dimensional measure, individual factor loadings are not necessarily high (Ćirović & Pedović, 2025). As such, network models based on individual conspiracy mentality items might lead to novel insights.

The present study investigates the relationship between conspiracy mentality and personality aspects examined extensively in the past, as well as variables that were largely ignored in the context of conspiracy thinking. The group of extensively discussed correlates of conspiracy beliefs consists of political cynicism, neuroticism, self-efficacy, self-esteem, and control beliefs. Since the average sign and strength of the relationship with conspiracy beliefs are known, the present findings can be directly compared to previous findings. Furthermore, bedtime procrastination and life satisfaction will also be investigated in the current study, adding to the growing knowledge about the correlates of conspiracy beliefs. The potential relevance of these constructs to conspiracy thinking is discussed in the following paragraphs.

### Political Cynicism

Political cynicism is usually positively related to conspiracy beliefs (Swami, 2012; Swami et al., 2011). People who hostilely distrust politicians with regard to their motives and actions tend to question official explanations of events, which might result in endorsing conspiracy theories postulating the involvement of politicians in “covering up” evidence related to real-life events.

### Life Satisfaction

Life satisfaction might be negatively related to conspiracy beliefs (Swami et al., 2011); poor life satisfaction might result from feeling disadvantaged, and it has been speculated that underprivileged people are more likely to endorse conspiracy theories. However, the findings are mixed (Swami, 2012; Swami et al., 2011). Examining the intricate relationships at the item level in the present study could shed light on this relationship; it cannot be ruled out that only certain aspects of life satisfaction are related to conspiracy beliefs.

### Self-Appraisal (Neuroticism, Self-Esteem, Self-Efficacy, and Locus of Control)

Several different constructs included in the present study are concerned with the self-appraisal of a person (Judge et al., 2002). High neuroticism, low self-esteem, low self-efficacy, high external locus of control, and low internal locus of control are generally thought to reflect a negative self-view. On the surface level, it might appear unnecessary to include several self-appraisal constructs. However, as shown in the following paragraphs, the strength of the relationship with conspiracy beliefs differs between the individual personality traits.

According to meta-analytic findings, a small negative relationship between self-esteem and conspiracy beliefs is usually reported. For example, Bowes and colleagues (2023) reported an average correlation of *r* = -.09, 95% CI [-.14, -.04]. Biddlestone and colleagues (2025) observed a correlation of *r* = -.10, 95% CI [ -.20, .01], and Stasielowicz (2022b) found a correlation of *r* = -.06, 95% CR [-.11, <.00]. Theoretically, this relationship could be bidirectional. On the one hand, people with relatively low self-esteem may be vulnerable to conspiracy theories because they provide potential explanations or the opportunity to blame other entities, including politicians and institutions, for their own problems and failures. On the other hand, believing in conspiracy theories could lead to a negative self-evaluation of one's ability to deal with problems. Exposure to conspiracy theories postulating the existence of uncontrollable forces or actors with bad intentions could lead to low self-esteem with a pessimistic worldview that assumes that external forces make it impossible for ordinary citizens to change the environment.

For self-efficacy and neuroticism, negligible average correlations were found in meta-analyses. Specifically, the average correlations with neuroticism reported across meta-analyses are *r* = .05, 95% CI [.03, .07], *r* = .03, 95% CI [-.02, .09], and *r* = .04, 95% CR [.01, .07] (Bowes et al., 2023; Goreis & Voracek, 2019; Stasielowicz, 2022b). For self-efficacy, Bowes and colleagues (2023) reported a correlation of *r* = -.07, 95% CI [-.22, .09] with substantial heterogeneity, indicating that self-efficacy might be relevant under certain circumstances.

Finally, people with low perceived control tend to believe in conspiracy theories (Biddlestone et al., 2025; Bowes et al., 2023). Whereas Bowes and colleagues found an average correlation of *r* = -.17, 95% CI [-.21, -.12], Biddlestone and colleagues reported a meta-analytic correlation of *r* = -.20, 95% CI [-.25, -.15] with external control and a correlation of *r* = -.04, 95% CI [-.11, .03] with personal control. The present study also distinguishes between control dimensions to enable the investigation of differential relationships with conspiracy mentality. Similar to self-esteem, a bidirectional relationship between perceived control and conspiracy mentality is plausible. People consuming conspiracy theories might conclude that politicians, health institutions, and other entities control many areas of everyday life (i.e., high external locus of control) and that their own actions do not matter (i.e., low internal locus of control). However, it is also possible that people who already have a low sense of control are attracted to conspiracy theories, as they offer the opportunity to blame others for the perceived lack of control.

### Bedtime Procrastination

Conspiracy mentality might be positively related to the tendency to go to bed later than intended (Kroese et al., 2014). Such bedtime delays can partially result from consuming online content in the evening. Ensuing sleep deficiency and associated symptoms, such as concentration difficulties, might make people more vulnerable to conspiracy theories disseminated on the Internet that bedtime procrastinators are likely to encounter while searching for exciting online content at night.

Overall, the present study seeks to:

- Examine to what extent the strength and sign of the relationship between personality variables and conspiracy mentality remain stable across bivariate and multivariate analyses (i.e., network models consisting of multiple personality variables).
- Investigate whether network models based on individual scale items offer novel insights when compared with models based on aggregate scale scores
- Examine the stability of networks across different external circumstances (before vs. during the COVID-19 pandemic).
- Identify potential targets for interventions addressing conspiracy beliefs.

## Method

### Sample Size Planning

Given the present research’s goal of identifying potential intervention targets, a threshold had to be set to decide what constitutes a promising intervention target. As various undergraduate projects were conducted as part of the current study, a simple correlation criterion was used. The correlation with conspiracy mentality had to be greater than a typical correlation between personality variables and conspiracy beliefs. Since the average correlation between conspiracy beliefs and previously examined personality variables is close to *r* = . 15 (Biddlestone et al., 2025), the threshold was set to *r* = .20. To achieve a power of 80% at this correlation threshold, a sample size of approximately 200 participants is required (R package *pwr2ppl* 0.5.0). Therefore, it was planned to collect data from at least 200 participants, with no upper limit to enable more complex analyses.

### Procedure

Users of internet forums and social media groups discussing conspiracy theories were asked to participate in a German online survey. In addition, other groups (primarily students) were approached to increase variability in the conspiracy mentality. Data were collected in two waves, before and during the COVID-19 pandemic (2019 and 2020, respectively), providing the unique opportunity to investigate network stability across different circumstances. To mitigate potential data dependency issues, different groups (e.g., student groups and internet forums) were contacted at each wave. In total, 673 people answered at least one question (1^st^ wave – 438 people, 2^nd^ wave – 235 people).

The project was carried out in accordance with the ethical guidelines of the American Psychological Association. After providing informed consent, participants received demographic questions. Only participants older than 18 received further questions. Specifically, these participants completed a few questionnaires for different undergraduate and graduate projects: Bedtime procrastination, neuroticism, self-esteem, life satisfaction, self-efficacy, political cynicism, locus of control (only during the second recruitment wave), conspiracy beliefs, and conspiracy mentality. Finally, participants could choose the compensation for completing the survey: (1) Draw (eight vouchers each worth 20 €), (2) Course credit, or (3) No compensation.

### Measures

Descriptive statistics of the relevant questionnaires are presented in Table 1. All relevant instruments had good or very good reliability across all samples.

**Table 1:** Descriptive Statistics (Metric Variables) for the Pre-Pandemic Sample (2019), Pandemic Sample (2020), and Pooled Data

	2019 Sample / 2020 Sample / Pooled Data				
Variable	*M*	*SD*	Min	Max	α [CR 95%]
Age	28.43	12.24	18	81	
	25.91	9.03	18	73	
	27.61	11.36	18	81	
Bedtime procrastination	3.08	0.91	1.00	5.00	.90 [.88, .91]
	3.17	0.89	1.00	4.89	.89 [.87, .92]
	3.11	0.91	1.00	5.00	.90 [.88, .91]
Conspiracy mentality	5.86	2.00	1.40	11.00	.85 [.83, .87]
	5.34	1.85	1.20	11.00	.84 [.80, .87]
	5.70	1.97	1.20	11.00	.85 [.83, .87]
Control (external)	—	—	—	—	—
	2.56	0.77	1.00	5.00	.76 [.71, .82]
	—	—	—	—	—
Control (internal)	—	—	—	—	—
	3.76	0.83	1.00	5.00	.88 [.85, .90]
	—	—	—	—	—
Life satisfaction	4.77	1.13	1.20	7.00	.84 [.82, .87]
	4.85	1.08	1.80	7.00	.84 [.80, .87]
	4.79	1.11	1.20	7.00	.84 [.82, .86]
Neuroticism	2.69	0.91	1.00	5.00	.86 [.84, .88]
	2.72	0.85	1.17	5.00	.83 [.79, .87]
	2.70	0.89	1.00	5.00	.85 [.83, .87]
Political cynicism	3.05	0.69	1.25	4.88	.83 [.80, .85]
	2.83	0.74	1.38	5.00	.84 [.80, .87]
	2.98	0.72	1.25	5.00	.83 [.81, .85]
Self-efficacy	2.83	0.46	1.30	4.00	.87 [.85, .88]
	2.84	0.45	1.50	4.00	.86 [.83, .89]
	2.83	0.45	1.30	4.00	.86 [.85, .88]
Self-esteem	3.17	0.61	1.20	4.00	.90 [.89, .92]
	3.21	0.58	1.40	4.00	.90 [.88, .92]
	3.19	0.60	1.20	4.00	.90 [.89, .91]

*Note. N*_2019_ = 403, *N*_2020_ = 193, *N*_pooled_ = 596; α = Reliability (Bayesian Cronbach’s alpha); CR = credibility interval limits (95%).

*Bedtime procrastination* was assessed with the Bedtime Procrastination Scale (Kroese et al., 2014). Participants completed all nine items (e.g., “Often I am still doing other things when it is time to go to bed”) using a five-point scale (1 – Almost never, 5 – Almost always).

*Conspiracy mentality* was measured with the Conspiracy Mentality Questionnaire (Bruder et al., 2013). The scale consists of five items (e.g., “I think that there are secret organisations that greatly influence political decisions”), and the responses are provided on an 11-point scale (1 = Certainly not, 11 = Certain).

*Life satisfaction* was assessed using the Satisfaction with Life Scale (Glaesmer et al., 2011). Participants responded to five items (e.g., “In most ways my life is close to my ideal”) using a seven-point scale (1 = Does not apply at all, 7 = Applies completely).

*Locus of control* was assessed only in the second sample. To this end, the two-ways-questionnaire for the assessment of control beliefs was used (Maes & Schmitt, 1999). Specifically, the internal locus of control (e.g., “Everyone has their life in their own hands”) and external locus of control (e.g., “Others have a decisive influence on your own life”) were assessed by four items using a five-point scale (1 = Completely disagree, 5 = Completely agree).

*Neuroticism* was measured using one subscale of the NEO−Five−Factor Inventory (Körner et al., 2008). Participants completed the relevant six items (e.g., “Sometimes I feel completely worthless”) using a five-point scale (1 = Completely disagree, 5 = Completely agree).

*Political cynicism* was assessed with the Political Cynicism Scale (Pattyn et al., 2012). Responses to the eight items (e.g., “Our political leaders are prepared to lie to us whenever it suits their purposes”) were provided using a five-point scale (1 = Completely disagree, 5 = Completely agree).

*Self-efficacy* was measured using the General Self-Efficacy Scale (Schwarzer & Jerusalem, 1995), which consists of ten items (e.g., “Thanks to my resourcefulness, I know how to handle unforeseen situations”). The responses were provided using a four-point scale (1 = Not true at all, 4 = Exactly true).

*Self-esteem* was assessed using the Rosenberg Self-Esteem Scale (von Collani & Herzberg, 2003). Participants completed the ten items (e.g., “I certainly feel useless at times”) using a four-point scale (1 = Does not apply at all, 4 = Applies completely).

### Sample

Seventy-seven out of 673 participants did not complete the survey or provided low-quality data (see Stasielowicz, 2024b for all exclusion criteria). Overall, before the COVID-19 pandemic, 403 participants could be included. During the pandemic, data from 193 participants were included. Since skipping questions was not possible in the online survey, there were no missing data in the final sample.

Most participants were female (Sample 1: 277 women, 123 men, and three intersex/diverse people; Sample 2: 140 women, 52 men, and one intersex/diverse person). The age varied between 18 and 81 (Sample 1: *Mdn* = 23, *M* = 28.43, *SD* = 12.24; Sample 2: *Mdn* = 23, *M* = 25.91, *SD* = 9.03).

### Statistical Analysis

#### Bivariate Correlations

To estimate the bivariate correlations between conspiracy mentality and personality variables, the Bayesian estimation approach was employed. An important advantage of the Bayesian approach is that it incorporates prior knowledge during modeling (Kruschke & Liddell, 2018), which can help mitigate convergence issues, particularly when dealing with complex models. The respective prior specifications are provided in the analysis scripts at Stasielowicz (2026b). For example, when estimating bivariate correlations between conspiracy mentality and personality variables using the *brms* package (2.19.0), the Lewandowski-Kurowicka-Joe distribution with a scale parameter of 2 was used [LKJ(2)] to incorporate the prior information that in social sciences, very high correlations are less likely to be found than smaller correlations.

The prior knowledge is combined with collected data, assuming a Gaussian likelihood, to yield the posterior distribution, which contains the most plausible results (e.g., correlation estimates). To capture potential problems during the estimation process, autocorrelation of the emerging posterior values was evaluated. High autocorrelation would signal that posterior values recorded later depend strongly on earlier posterior values, which in turn could mean that the space of possible results is not sufficiently explored during the estimation process, and the model needs to be revised.

Based on the relevant posterior distributions (e.g., the most plausible estimates of the correlation between variables), a point estimate is provided, along with an uncertainty interval, throughout the manuscript. The 95% credible intervals (CR) are computed as the 2.5^th^ and 97.5^th^ percentiles of the posterior distribution.

#### Network Models

Bayesian network analyses were conducted to examine the relationship between the aggregate scores and individual items of conspiracy mentality and other constructs (R package *BGGM 2.0.4*). The present manuscript follows the recently proposed reporting standards for network analyses (Burger et al., 2023).

All variables were coded as continuous in the network model when investigating the relationships between mean scale scores. However, only life satisfaction and conspiracy mentality items were coded as continuous variables when examining the relationships between individual items. The other items were coded as ordinal because few response options were available (e.g., a four-point scale). All reverse-coded items were inverted to enable consistent interpretation across the items belonging to the same scale.

The *BGGM* package uses a Beta distribution centered at zero as the prior for partial correlations in network models, allowing users to specify the standard deviation of the distribution. The default standard deviation parameter of 0.25 was used because very high partial correlations are less likely than smaller values. Whereas Gaussian networks were used for aggregate scores, ranked likelihood (Williams & Mulder, 2020) was used for ordinal variables in the item-level models. As in bivariate analyses, autocorrelation was evaluated to capture potential estimation problems.

To identify the central network elements, betweenness centrality, closeness centrality, strength centrality, and expected influence indices were inspected for each network node (Costantini et al., 2015). Betweenness centrality refers to the number of shortest paths between other network nodes passing through a specific node. Closeness centrality reflects the shortest path lengths between a particular node and all other nodes. Strength centrality indicates the absolute sum of the strength of the connections (edges) with other network nodes. In contrast, expected influence reflects whether the sign of the relationship with other nodes is consistent; positive and negative relationships cancel each other out, leading to expected influence values closer to zero (Robinaugh et al., 2016). As shown by Robinaugh and colleagues, information about centrality indices can be helpful when preparing future interventions. For example, it may be worth targeting the most influential conspiracy mentality item, especially if the particular conspiracy mentality aspect appears amenable.

To facilitate assessing the stability of the networks in the present study, the main analyses were conducted separately using the combined data (2019 and 2020), the 2019 data, the reduced 2020 data (without locus of control), and the complete 2020 data (with locus of control). This approach enables investigating whether the network structure is stable across different external circumstances (i.e., during and before a worldwide pandemic) and when additional variables are included (i.e., 2020 data with and without locus of control items).

As for bivariate correlations, point estimates, along with 95% credible intervals, are reported for network parameters throughout the manuscript to acknowledge the uncertainty of these estimates. All specifications, data, R code, and output are available at Stasielowicz (2026b).

## Results

### Descriptive Statistics

**Figure 1:**
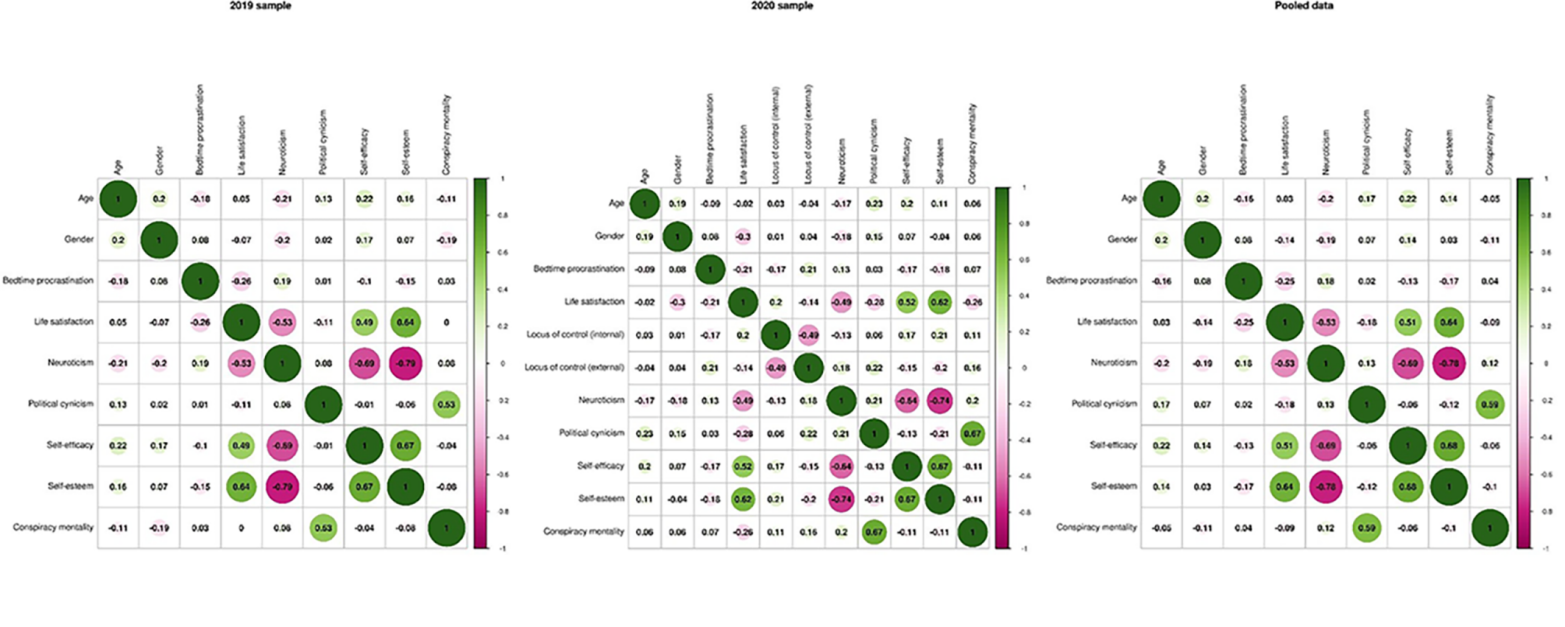
Bayesian Bivariate Correlations Between Demographic Variables, Conspiracy Mentality, and Other Questionnaires *Note.* (*N*_2019_ = 403, *N*_2020_ = 193, *N*_pooled_ = 596). Because of the small group size, correlations with gender (0 = Female, 1 = Male) do not include the four people identifying as Intersex/Diverse (*N*_2019_ = 400, *N*_2020_ = 192, *N*_pooled_ = 592).

The descriptive statistics of the main questionnaires are reported separately for both measurement waves and the combined sample in Table 1. Bayesian bivariate correlations between demographic variables and questionnaire scores are presented in Figure 1.

Despite the different external circumstances, both the pre-pandemic and pandemic samples showed similar patterns. For example, there were strong bivariate relationships among the three traditional self-appraisal constructs (i.e., neuroticism, self-efficacy, and self-esteem). Notably, only political cynicism was strongly related to conspiracy mentality across both measurement waves. Interestingly, during the pandemic, conspiracy mentality was more strongly related to life satisfaction and neuroticism than before the pandemic. Specifically, people with relatively low life satisfaction or high neuroticism reported a higher conspiracy mentality. In addition, people with a high external locus of control, which was only assessed in the second sample, tended to have a high conspiracy mentality.

To examine whether relationships hold up when simultaneously accounting for multiple variables, network analyses with mean questionnaire scores were conducted in the next step.

### Network Analysis (Mean Scores)

#### Partial Correlations

**Figure 2:**
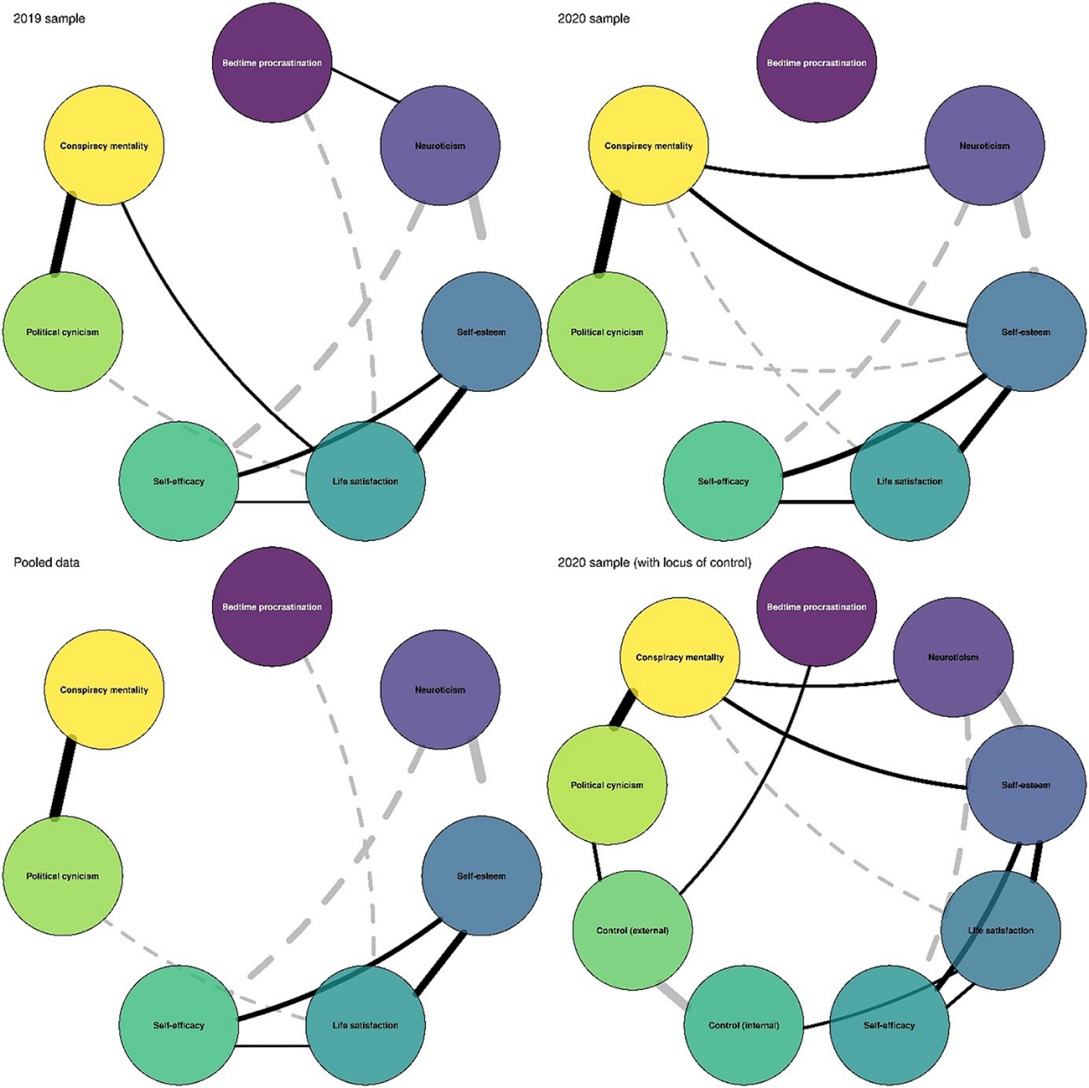
Network Models (*N*_2019_ = 403, *N*_2020_ = 193, *N*_pooled_ = 596) Consisting of Conspiracy Mentality and Various Personality Mean Scores *Note.* The width of the connection (edges) represents the strength of the relationship (partial correlation). solid black curves represent positive relationships, and gray dashed curves represent negative relationships. The variables are arranged in a circle to facilitate network comparisons.

Figure 2 contains network visualizations for the respective samples. A systematic comparison of the pre-pandemic and pandemic networks indicated that the networks cannot be regarded as equal. Although the observed correlation between the networks (i.e., the relationship between the partial correlation matrices of the two networks)

was high (*r* = .83), this value did not overlap substantially with the predictive distribution assuming network equality, CR 95% [.88, .98].

Still, Figure 2 indicates that multiple relationships were stable across both samples. Even after accounting for additional variables, political cynicism was strongly related to conspiracy mentality in both samples, 2019: .54 CR 95% [.47, .61]; 2020: .66 CR 95% [.57, .73].

A few noteworthy relationships contributing to network inequality primarily involve conspiracy mentality. While before the pandemic, there was a small positive relationship between life satisfaction and conspiracy mentality, during the pandemic, a small negative relationship was found, 2019: .14 CR 95% [.05, .24]; 2020: -.15 CR 95% [-.29, <.00]. Furthermore, only during the pandemic neuroticism, 2019: .00 CR 95% [-.10, .10]; 2020: .17 CR 95% [.02, .31] and self-esteem, 2019: -.09 CR 95% [-.19, .01]; 2020: .21 CR 95% [.07, .35] were positively related to conspiracy mentality. Importantly, the network for the pandemic data did not change considerably after locus of control variables were included (see Figure 2).

#### Shared Variance (*R*^2^)

Across both samples, the node that shared the most variance (*R*^2^) with other variables was self-esteem, 2019: .72 CR 95% [.65, .80]; 2020: .72 CR 95% [.62, .83]. The node that shared the least variance was bedtime procrastination, 2019: .10 CR 95% [.05, .15]; 2020: .09 CR 95% [.03, .16].

As already indicated by the partial correlations, conspiracy mentality shared more variance with other constructs during the pandemic than before the pandemic, 2019: .31 CR 95% [.25, .38]; 2020: .51 CR 95% [.41, .61]. Again, the estimates did not change considerably when the locus of control variables were included in the 2020 sample.

#### Centrality Measures

Since the uncertainty around the estimates of betweenness and closeness centrality is relatively large, they are reported primarily for the sake of transparency. It is recommended to focus on strength and expected influence when comparing centrality indices of different network elements in the present study.

Interestingly, the highest expected influence was found across all samples for conspiracy mentality, 2019: 0.63 CR 95% [0.39, 0.86]; 2020: 0.94 CR 95% [0.58, 1.29] and neuroticism, 2019: -0.71 CR 95% [-0.89, -0.53]; 2020: -0.70 CR 95% [-0.97, -0.42]. This pattern indicates that successfully changing conspiracy beliefs might have broad implications for various personality constructs. In contrast, the expected influence of bedtime procrastination was relatively low across both samples, 2019: 0.05 CR 95% [-0.17, 0.28]; 2020: -0.26 CR 95% [-0.57, 0.06].

Before the pandemic, conspiracy mentality was slightly closer (i.e., shorter paths) to other constructs than during the pandemic, 2019_closeness_: 0.02 CR 95% [0.01, 0.03]; 2020_closeness_: 0.03 CR 95% [0.02, 0.04]. The closeness centrality values were similar to those of bedtime procrastination, which tended to be the closest to other constructs, 2019_closeness_: 0.02 CR 95% [0.02, 0.03]; 2020_closeness_: 0.02 CR 95% [0.01, 0.03]. Self-esteem tended to have the longest paths, 2019_closeness_: 0.03 CR 95% [0.02, 0.04]; 2020_closeness_: 0.04 CR 95% [0.03, 0.05].

The highest values of the other two centrality indices (i.e., betweenness and strength centrality) were usually found for self-esteem, 2019_betweenness_: 4.87 CR 95% [1.00, 8.00]; 2020_betweenness_: 5.67 CR 95% [1.00, 11.00], 2019_strength_: 1.42 CR 95% [1.21, 1.66]; 2020_strength_: 1.63 CR 95% [1.31, 1.96]. In the pandemic data, conspiracy mentality had a relatively high number of the shortest paths between other network variables passing through it (i.e., betweenness) and a relatively high absolute strength of the connections with personality variables, 2019_betweenness_: 0.58 CR 95% [0.00, 5.00]; 2020_betweenness_: 2.81 CR 95% [0.00, 6.00], 2019_strength_: 0.91 CR 95% [0.70, 1.16]; 2020_strength_: 1.33 CR 95% [0.98, 1.67]. These centrality estimates were usually considerably smaller for bedtime procrastination, 2019_betweenness_: 0.02 CR 95% [0.00, 0.00]; 2020_betweenness_: 0.01 CR 95% [0.00, 0.00], 2019_strength_: 0.61 CR 95% [0.35, 0.90]; 2020_strength_: 0.49 CR 95% [0.24, 0.82].

### Network Analysis (Individual Items)

Since focusing on the relationships between aggregate scores might mask intricate patterns, network models consisting of individual questionnaire items were built in the next step. Because of model complexity (e.g., the number of items, ordinal data), the uncertainty around the estimates is large for these models. The uncertainty is additionally increased by the reduced willingness to participate in surveys early during the COVID-19 pandemic (e.g., taking care of children during lockdowns had higher priority). Therefore, unless otherwise stated, rather than directly comparing the pre-pandemic and pandemic data, pre-pandemic data were compared to the pooled data to determine whether the relationships identified in the pre-pandemic data changed considerably after including data collected during the pandemic.

Although most of the identified item-level relationships were plausible (e.g., the sign of the relationship), and the respective estimates might help inform future hypothesis-based research, it would be too speculative to provide recommendations for designing interventions at this stage. Therefore, a cautious interpretation of the item-level patterns is provided for interested readers in the Open Repository at Stasielowicz (2025).

## Discussion

The present study applied network modeling across different samples to offer a nuanced perspective on the relationship between various personality variables and conspiracy mentality. Specifically, the relationships between bedtime procrastination, life satisfaction, locus of control, neuroticism, political cynicism, self-efficacy, self-esteem, and conspiracy mentality were examined at the item level and aggregate score level before and during the COVID-19 pandemic, which provided the unique opportunity to investigate the stability of the networks across different external circumstances.

With regard to the main objectives, the present study found, (a) that while the strength and sign of the relationship between personality variables and conspiracy mentality appears to be stable for various relationships across bivariate analyses and network models (i.e., after accounting for other personality variables), the latter approach leads to some novel insights, (b) that although many network relationships are stable across different external circumstances (before vs. during the COVID-19 pandemic), there are some noteworthy exceptions which may indicate that interventions targeting implausible conspiracy beliefs need to be tailored to the external circumstances, and (c) that network models may help identify influential variables and potential intervention targets.

### Bivariate Correlations vs. Network Models

Overall, the network models based on aggregate scores paint a slightly different picture than the bivariate correlations (see Figure 1 and Figure 2). While both bivariate and partial correlations confirmed that political cynicism tends to go along with a high conspiracy mentality, the relationships with life satisfaction, self-esteem, and external locus of control were different. In the pre-pandemic data, the negligible bivariate correlation between conspiracy mentality and life satisfaction became positive after accounting for various personality variables in the network model. Furthermore, in the network model based on the pandemic data, a negligible negative relationship with self-esteem turned into a small positive relationship, and the positive relationship with an external locus of control became negligible.

Because of the differences between bivariate correlations and partial correlations from network models, one should not assume that the magnitude or even sign of the causal relationship with antecedents and consequences of conspiracy beliefs can be derived from the meta-analytic estimates of bivariate correlations (Biddlestone et al., 2025; Bowes et al., 2023; Hornsey et al., 2023; Stasielowicz, 2022b). At the same time, it is worth noting that cross-sectional network models do not satisfy all three typical social science causality criteria (Preacher, 2015). Although cross-sectional network models can help, (a) establish a relationship between two variables, and (b) rule out alternative explanations by adjusting for further variables, the (c) temporal causality condition is not met. Therefore, it is recommended to conduct longitudinal network analyses to ensure that the presumed cause is assessed before the consequence, increasing the plausibility of a causal relationship.

### Network Stability

The unexpected start of a pandemic before the second data collection enabled the investigation of the stability of psychometric networks. Despite the radically different external circumstances (e.g., pandemic measures such as lockdowns and social distancing), many network relationships were stable across the samples, including the positive relationship between political cynicism and conspiracy mentality and the negligible relationship between self-efficacy and conspiracy mentality.

Still, a test systematically comparing the networks revealed that, despite the high correlation, the networks cannot be regarded as equal. For example, before the COVID-19 pandemic, life satisfaction was positively related to conspiracy mentality, whereas during the pandemic, there was a negative relationship. The data collection strategies and sample characteristics cannot explain this pattern because they were similar across both samples (see Table 1). However, Germany implemented strict restrictions, such as mask-wearing mandates and social distancing protocols, in the first years of the pandemic. It is possible that during the pandemic, people with a high conspiracy mentality had relatively low life satisfaction because they were frustrated by the wide-ranging preventive measures they attributed to the purported ongoing conspiracies. In contrast, before the pandemic, a high conspiracy mentality corresponded to higher life satisfaction, which could mean that conspiracy theories offer consolation by providing explanations for events in relatively peaceful times.

The observed pattern of relationships changing across different circumstances may have implications for the development of interventions. Amenable intervention targets may be different during crises. Paying attention to citizens’ life satisfaction, especially during crises, could reduce vulnerability to implausible conspiracy beliefs. However, these novel insights need to be confirmed in future studies comparing the stability of networks before, during, and after political, societal, and economic crises.

Item-based networks were somewhat less stable than networks based on aggregate scores. On the one hand, it could mean that some item-level relationships are spurious. On the other hand, it cannot be ruled out that model complexity (e.g., number of estimated relationships, ordinal data) contributed to the uncertainty of the estimates. Furthermore, similar to the aggregated data, some sample differences might stem from the varying external circumstances.

Notably, various additional comparisons indicated that networks were relatively stable. For example, aggregate score networks did not change considerably after the locus of control variables were included. Finally, the rank order and estimated magnitude of the centrality indices (i.e., betweenness, closeness, strength, and expected influence) were often similar across samples.

### Potential Intervention Routes

Identifying novel intervention routes is important as available interventions usually have a small impact on conspiracy beliefs (O’Mahony et al., 2023; Stasielowicz, 2026a). Since bivariate analyses and mean-score-based network models across all samples confirmed a positive relationship between political cynicism and conspiracy mentality (Swami, 2012; Swami et al., 2011), it may be worth examining whether increasing the politicians’ transparency and accountability (e.g., compulsory lobby registers) could help reduce the conspiracy mentality. Future studies could use databases with transparency rankings to investigate whether countries with higher transparency and accountability tend to have a lower conspiracy mentality.

Finally, centrality measures in both samples indicated that conspiracy mentality had the highest expected influence in the networks based on mean scores. Thus, reducing conspiracy mentality might impact various personality variables. Such hints are important as the causal effects of conspiracy beliefs are not well-established; intervention studies focus on reducing conspiracy beliefs without examining downstream consequences (Stasielowicz, 2026a).

### Limitations

Although sample composition was similar before and during the COVID-19 pandemic, which enabled assessing the robustness of the findings in similar groups, most of the 600 participants were relatively young (*Mdn* = 23, Min = 18, Max = 81) compared to the median age in Germany - 47 (Ritchie & Roser, 2019). Furthermore, twice as many women as men participated in the current study, which differs from the balanced ratio in the general population.

As indicated by the uncertainty of some network estimates, especially at the item level, the power for some analyses was not optimal. Sample size planning was primarily conducted with bivariate correlation analyses in mind for undergraduate projects. While the target sample size for those analyses could be reached, more participants are required for more complex analyses. To reduce the uncertainty of the estimates, researchers are encouraged to conduct large-scale studies, preferably as multi-lab projects, to ensure the project's feasibility.

For practical reasons, the present study could not consider all correlates of conspiracy beliefs examined in meta-analyses (Biddlestone et al., 2025; Bowes et al., 2023; Hornsey et al., 2023; Stasielowicz, 2022b). Including constructs such as narcissism, paranoia, schizotypy, and religiosity could lead to other fine-grained implications in future studies.

Finally, the current study's cross-sectional design and unrestricted time frame in the questionnaires’ instructions cannot be used to derive causal conclusions. It is plausible that personality acts both as a cause and an outcome. Furthermore, similar to the bidirectional relationship between conspiracy beliefs and COVID-19-related preventive behavior (Stasielowicz, 2022a), it cannot be ruled out that the strength of the relationships examined in the present study depends on the time lag between assessing the respective constructs. Furthermore, although mostly stable personality chararcteristics were assessed in the present study, which were thought to occupy a similar place in the causal chain, it cannot be ruled out that there are collider-type relationships which may bias correlation estimates (Cinelli et al., 2024; Rohrer, 2018) or that relevant variables were not included in the networks. Future studies will benefit from incorporating theoretical deliberations about the causal order of the variables and the meta-analytic evidence about the strength of the relationships, using longitudinal designs accounting for variables regarded as relevant predictors of conspiracy beliefs (Biddlestone et al., 2025; Bowes et al., 2023; Hornsey et al., 2023; Stasielowicz, 2022b) and using time lags between three and six months. Following these recommendations may help establish whether the relationship between conspiracy mentality and personality is bidirectional and how it evolves over time.

## Supplementary Materials

**Table:** 

Type of supplementary materials	Availability/Access
Data	
Study data.	Stasielowicz (2026b)
Code	
Scripts and output.	Stasielowicz (2026b)
Material	
Figures.	Stasielowicz (2026b)
Script outputs.	Stasielowicz (2026b)
Item-level summary.	Stasielowicz (2026b)
Study exclusion criteria.	Stasielowicz (2024b)
Study/Analysis preregistration	
The study was not preregistered.	—
Other	
Study supplement interpreting item-level patterns.	Stasielowicz (2025)

## Data Availability

The datasets generated and/or analyzed during the current study are available in the OSF repository at Stasielowicz (2026b).
